# The Association Between Increased Maladaptive Health Behaviours and Elevated Mental Health Symptoms Among Persons with IBD During the COVID-19 Pandemic

**DOI:** 10.1093/jcag/gwad030

**Published:** 2023-09-13

**Authors:** Casandra L Dolovich, Seth R Shaffer, Lesley A Graff, Harminder Singh, Renée El-Gabalawy, Souradet Shaw, Charles N Bernstein

**Affiliations:** Department of Internal Medicine, Max Rady College of Medicine, Rady Faculty of Health Sciences, University of Manitoba, C4-820 Sherbrook St Winnipeg, Manitoba, R3A1R9Canada; University of Manitoba IBD Clinical and Research Centre, University of Manitoba, MS-783 Sherbrook St Winnipeg, Manitoba, R3A1R9Canada; Department of Internal Medicine, Max Rady College of Medicine, Rady Faculty of Health Sciences, University of Manitoba, C4-820 Sherbrook St Winnipeg, Manitoba, R3A1R9Canada; University of Manitoba IBD Clinical and Research Centre, University of Manitoba, MS-783 Sherbrook St Winnipeg, Manitoba, R3A1R9Canada; University of Manitoba IBD Clinical and Research Centre, University of Manitoba, MS-783 Sherbrook St Winnipeg, Manitoba, R3A1R9Canada; Department of Clinical Health Psychology, Max Rady College of Medicine, Rady Faculty of Health Sciences, University of Manitoba, PZ-350 820 Sherbrook St Winnipeg, Manitoba, R3A1R9Canada; Department of Internal Medicine, Max Rady College of Medicine, Rady Faculty of Health Sciences, University of Manitoba, C4-820 Sherbrook St Winnipeg, Manitoba, R3A1R9Canada; University of Manitoba IBD Clinical and Research Centre, University of Manitoba, MS-783 Sherbrook St Winnipeg, Manitoba, R3A1R9Canada; University of Manitoba IBD Clinical and Research Centre, University of Manitoba, MS-783 Sherbrook St Winnipeg, Manitoba, R3A1R9Canada; Department of Clinical Health Psychology, Max Rady College of Medicine, Rady Faculty of Health Sciences, University of Manitoba, PZ-350 820 Sherbrook St Winnipeg, Manitoba, R3A1R9Canada; Department of Internal Medicine, Max Rady College of Medicine, Rady Faculty of Health Sciences, University of Manitoba, C4-820 Sherbrook St Winnipeg, Manitoba, R3A1R9Canada; University of Manitoba IBD Clinical and Research Centre, University of Manitoba, MS-783 Sherbrook St Winnipeg, Manitoba, R3A1R9Canada; Department of Internal Medicine, Max Rady College of Medicine, Rady Faculty of Health Sciences, University of Manitoba, C4-820 Sherbrook St Winnipeg, Manitoba, R3A1R9Canada; Department of Internal Medicine, Max Rady College of Medicine, Rady Faculty of Health Sciences, University of Manitoba, C4-820 Sherbrook St Winnipeg, Manitoba, R3A1R9Canada; University of Manitoba IBD Clinical and Research Centre, University of Manitoba, MS-783 Sherbrook St Winnipeg, Manitoba, R3A1R9Canada

**Keywords:** mental health, maladaptive behaviors, inflammatory bowel disease, pandemic, COVID-19

## Abstract

**Aim:**

To assess the association between maladaptive health behaviours and elevated mental health (MH) symptoms during the COVID-19 pandemic among persons with inflammatory bowel disease (IBD).

**Methods:**

Participants of the population-based University of Manitoba IBD Research Registry (*n* = 2,942) were invited to participate in a survey in November 2020, regarding their experiences during the COVID-19 pandemic. Maladaptive health behaviours included increased use of alcohol, marijuana, and cigarettes, and reduced exercise relative to pre-pandemic levels. Clinically significant MH symptoms were defined by the presence of elevated anxiety, depression, and/or post-traumatic stress. Adjusted logistic regression assessed the odds of elevated MH symptoms predicted by maladaptive health behaviours, stratified by gender.

**Results:**

Of 1,363 (46%) respondents, 319 (23%) had elevated MH symptoms. Those with elevated MH symptoms were older (mean age 54) and predominantly females (70%). The odds of any elevated MH symptoms were approximately two to four times greater among those who experienced maladaptive health behaviours during the pandemic including: increased alcohol use [aOR 2.14, 95% CI (1.50–3.05)], males who increased marijuana use [aOR 4.18, 95% CI (1.18–14.74)], females who increased smoking cigarettes [aOR 3.68 95% CI (1.15–11.86)] and any maladaptive health behaviour [aOR 1.93 95% CI (1.44–2.60)].

**Conclusion:**

During the COVID-19 pandemic, persons with IBD who experienced any maladaptive health behaviour was associated with double the likelihood of experiencing clinically significant MH symptoms. For persons with elevated MH symptoms, it is important for health care providers to recognize the association of increased maladaptive behaviours. Alternatively, if it is determined that MH symptoms predated maladaptive health behaviours then, inquiries into MH and providing appropriate referrals should be pursued.

## Introduction

It is known that persons with inflammatory bowel disease (IBD) have a greater prevalence of psychiatric comorbidity compared with the general population,^1^ such as anxiety and depression^2^ and post-traumatic stress disorder^3^ (PTSD). Early in the COVID-19 pandemic, there were already indications of a detrimental impact on mental health (MH), including for individuals with chronic diseases. For example, an Australian survey reported that among 45% of individuals with IBD and a pre-existing diagnosis of depression and/or anxiety, over two-thirds of these respondents reported worsening of their MH condition due to the pandemic.^4^ Addressing psychiatric comorbidity plays an important role in the health of persons with IBD, and failure to address these MH conditions may have a significant negative impact,^5^ such as worsened disease activity.^6^

Widespread stress has been significant during the COVID-19 pandemic, and consequently maladaptive health behaviours have become increasingly common. Reported increased rates of alcohol consumption, cannabis, and smoking during the COVID-19 pandemic have been estimated at about 20%–30%,^7,8^ along with reduced physical activity,^9^ which are further elevated among persons with MH conditions.^7,9^ Little research has been conducted regarding maladaptive health behaviours among persons with IBD and the relationship with MH symptoms during the COVID-19 pandemic.

As such, among those with IBD, we aimed to (1) determine the prevalence of current health behaviours among persons with/without elevated MH symptoms and (2) assess the relationship between maladaptive health behaviours and elevated MH symptoms such as anxiety, depression, and/or post-traumatic stress (PTS) during the COVID-19 pandemic.

## Methods

### Data source

Participants of the population-based University of Manitoba IBD Research Registry (*N* = 2,942) were invited in November 2020 to participate in a survey regarding their experiences during the COVID-19 pandemic, which in this central Canadian province included a first wave, and half way through the second wave around the timing of the survey. At which time, there were no approved vaccinations. Participants would have experienced lockdowns, social isolation, and the start of stricter restrictions such as mandating masks in all indoor places September 28, 2020.^10^ An invitation letter including a consent form, IBD COVID-19 survey, and an honorarium of $10 were mailed to the registry participants. The participants had the option of either completing the survey via paper copy or by accessing an online link. Depending on available contact information, reminder letters or e-mails were sent to those who had not responded between 2 and 4 weeks. The study was approved by the University of Manitoba Research Ethics Board. The survey included demographic and clinical information, validated measures for assessing MH symptoms as well as items regarding any changes in health behaviours such as use of alcohol, marijuana, cigarette smoking, and level of exercise.

### Demographic characteristics

Demographic characteristics included: age measured continuously and as a binary variable (17–64 years, age ≥ 65 years). The binary age variable was created to represent increased disease risk (of COVID-19) in older adults.^11^ Other demographics included gender (male, female), disease type [ulcerative colitis (UC), ileoanal pouch, Crohn’s disease (CD)], disease duration of IBD (0–10 years, 11–19 years, 30+ years), change in household income because of the COVID-19 pandemic, and use of any immune-modifying therapies (any of corticosteroids, azathioprine/6-mercaptopurine, methotrexate, biologic therapy including infliximab, adalimumab, vedolizumab, ustekinumab, or tofacitinib).

### Maladaptive health behaviours

Maladaptive health behaviours were defined as increased use of alcohol, marijuana, cannabidiol (CBD), and cigarette smoking, and reduced exercise during the COVID-19 pandemic. An example of survey questions pertaining to alcohol included “Do you drink alcohol,” and “If you answered yes that you drink alcohol, during the COVID-19 outbreak did you increase the number of drinks of alcohol per week that you had.” If the first question was affirmative, then a person was defined as a “current drinker.” If the second question was affirmative, a person was defined as having “increased intake of alcohol during COVID-19.” The other health behaviours were defined in a similar manner. For health behaviour exercise, the first question “Have you been regularly exercising since COVID-19?” defined a person as “regularly exercised” comparable to pre-pandemic times. The second question “If yes, how does this compare to how much you exercised before the COVID-19 pandemic”—“more,” “the same as usual,” or “less,” defined a person as having “reduced exercise during COVID-19” if they responded “less.” If a person increased any of alcohol, marijuana, CBD, cigarette smoking, or reduced exercise during the COVID-19 outbreak, we defined them as having “any maladaptive health behavior.”

### Mental health assessment—primary outcome(s)

MH symptoms were assessed using reliable and validated self-report measures for anxiety; Generalized Anxiety Disorders-7 (GAD),^12^ depression; Patient Health Questionnaire (PHQ-9),^13^ and PTS; PTSD checklist-5 (PCL-5).^14^ Total items and score ranges for the questionnaires were as follows: GAD-7 is made up of 7 items (total score ranging from 0 to 21), PHQ-9 is 9 items (total score ranging from 0 to 27), and PCL-5 consists of 20 items (total severity score ranging from 0 to 80). Clinically significant MH symptoms were defined using the following thresholds: GAD-7 and PHQ-9 scales ≥ 10^13,15^ for defining elevated symptoms of anxiety and depression, respectively, and PCL-5 scale ≥ 33^16^ suggesting elevated PTS symptoms. “Any clinically significant MH symptoms” was defined by a person having elevated symptoms in any 1 of the 3 scales. Clinically significant MH symptoms are referred to as “any elevated mental health symptoms.”

### Statistical analysis

Descriptive statistics were presented as mean and standard deviation for continuous variables and percentages for categorical variables. Since those with elevated MH symptoms were mostly females, we assessed whether there was a difference in proportions of maladaptive health behaviours for males compared with females among those with “any elevated mental health symptoms” using Fisher’s exact test. We conducted a bivariate analysis for maladaptive health behaviours (increased alcohol, marijuana, CBD, and cigarette smoking, and reduced exercise, or any maladaptive health behaviour) between elevated MH symptoms (anxiety, depression, PTS, any elevated symptom) compared to patients without any elevated MH symptoms using Fisher’s exact test. Lastly, adjusted logistic regression analysis was performed to predict the odds of “any elevated MH symptoms” by maladaptive health behaviours, stratified by gender. Models were adjusted for age ≥ 65 years, gender, disease type (UC, and ileoanal pouch versus CD), disease duration (11–19 years, 30+ years versus 0–10 years), change in household income because of the COVID-19 pandemic (yes versus no), and use of any immune-modifying therapies. All statistically significant results were presented as *P* < 0.05.

## Results

Of the 2,942 potential participants, there were 1,363 (46%) respondents. Respondents from the IBD Registry had similar demographic characteristics such as age, and disease type, compared with the non-respondents (Supplementary Table S1). However, the proportion of non-respondents was greater for males (45.9% versus 40.5%, *P* < 0.01), never-married persons (25.5% versus 8.9%, *P* < 0.0001), and current smokers (23.3% versus 8.9%, *P* < 0.0001).

Among respondents, 319 (23%) had clinically significant MH symptoms (Table 1). Persons with “any elevated MH symptoms” were predominantly females and younger compared with persons without elevated MH symptoms (69.9% versus 56.3%, *P* < 0.0001) and [mean SD; 54 (13.9) versus 59 (13.7), *P* < 0.0001], respectively. The proportions of persons with IBD and “any elevated MH symptoms” who smoked/ingested marijuana (18.7%), used CBD (14.5%), or smoked cigarettes (12.3%) were statistically significantly greater than the proportion of persons without “any elevated MH symptoms”; 12.7%, 8.7%, and 7.8%, respectively. There were no significant differences in the proportion of current alcohol drinkers between groups. The proportion of persons who regularly exercised during the pandemic to date (November 2020) was much lower among persons with “any elevated MH symptoms” (43.2%) compared to persons without “any elevated MH symptoms” (61.4%), *P* < 0.0001.

**Table 1.** Demographic characteristics and health behaviours, stratified by clinically significant mental health symptoms (*N* = 1,363).

	All respondents	Elevated mental health symptoms (anxiety, depression, and/or PTS)		*P*-value
	*N* = 1,363	No (*n* = 1,044)	Yes (*n* = 319)	
**Demographic characteristics, *n* (%)**	—	—	—	—
Age years, mean (SD)	58.1 (13.92)	59.3 (13.67)	54.0 (13.95)	<0.0001^*^
Age ≥ 65 years	480 (35.29)	398 (38.16)	82 (25.87)	<0.0001^*^
Females	811 (59.50)	588 (56.32)	223 (69.91)	<0.0001^*^
Disease type	—	—	—	0.2503
Crohn’s disease	647 (47.47)	489 (46.84)	158 (49.53)	—
Ulcerative colitis	686 (50.33)	535 (51.25)	151 (47.34)	—
Ileoanal pouch	30 (2.20)	20 (1.92)	10 (3.13)	—
Disease duration	—	—	—	0.2015
0–10 years	202 (15.87)	156 (16.00)	46 (15.44)	—
11–19 years	594 (46.66)	442 (45.33)	152 (51.01)	—
30+ years	477 (37.47)	377 (38.67)	100 (33.56)	—
Household income changed due to COVID-19^a^	346 (25.63)	243 (23.48)	103 (32.70)	0.0015^*^
Immune-modifying therapies	512 (37.56)	370 (35.44)	142 (44.51)	0.0037^*^
Health behaviours, *n* (%)	—	—	—	—
Currently drink alcohol	876 (64.79)	681 (65.86)	195 (61.32)	0.1403
Currently smoke, or ingest marijuana	190 (14.15)	131 (12.74)	59 (18.73)	0.0096^*^
Currently use cannabidiol	132 (10.02)	87 (8.65)	45 (14.47)	0.0047^*^
Currently a cigarette smoker	120 (8.87)	81 (7.82)	39 (12.30)	0.0175^*^
Regularly exercising	774 (57.12)	637 (61.37)	137 (43.22)	<0.0001^*^

^*^Statistically significant at *P* < 0.05, calculated using the Fisher’s exact test.

^a^Missing, *n* = 13.

**Figure 1.**
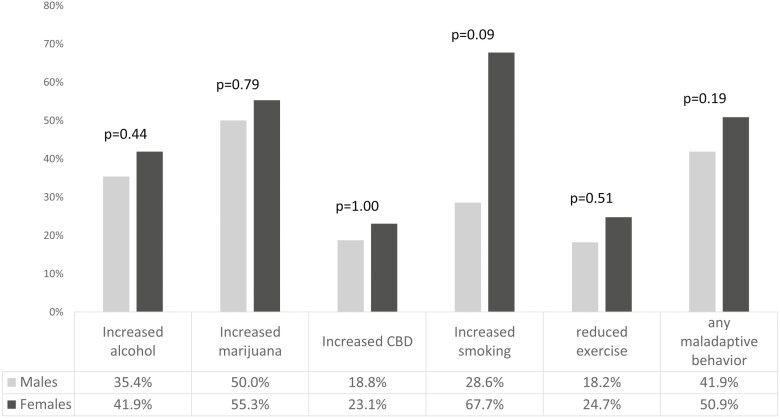
Proportion of maladaptive health behaviours among patients with any elevated mental health symptoms (anxiety, depression, or PTS), males versus females.

Among persons with “any elevated MH symptoms,” there were no differences in proportions of maladaptive health behaviours for males compared with females (fig. 1).

**Figure 2.**
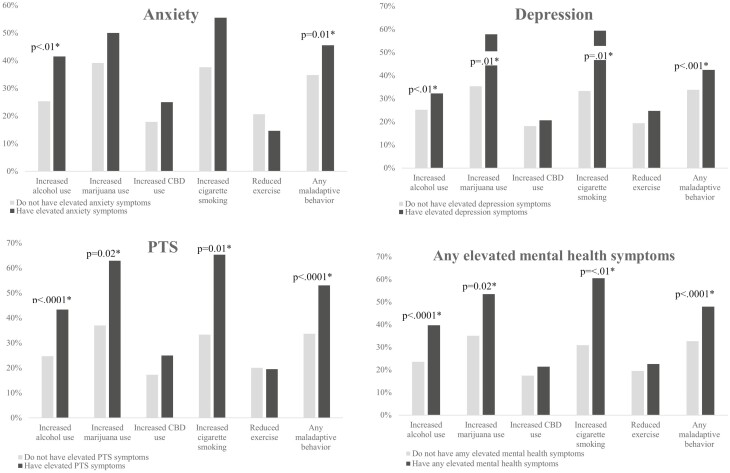
Proportion of maladaptive health behaviours, among patients with elevated mental health symptoms; anxiety, depression, PTS, compared with patients without any elevated mental health symptoms. *Statistically significant at *P* < 0.05, calculated using the Fisher’s exact test.

Figure 2 displays the bivariate analysis for maladaptive health behaviours between “any elevated MH symptoms” compared to persons without “any elevated MH symptoms.” Since we did not find any significant associations between gender and maladaptive health behaviours among elevated MH symptoms, we did not stratify results by gender. The proportion of increased alcohol use was greater for persons with elevated MH symptoms compared with the persons without elevated MH symptoms for anxiety (41.9% versus 25.4%, *P* < 0.01), depression (37.8% versus 25.2%, *P* < 0.01), PTS (43.4% versus 24.7%, *P* < 0.0001), and “any elevated MH symptom” (39.7% versus 23.6%, *P* < 0.0001). The proportion of increased marijuana use was greater among persons with elevated depression symptoms (57.8% versus 35.4%, *P* = 0.01), elevated PTS symptoms (63.0% versus 37.0%, *P* = 0.02), and “any elevated MH symptoms” (53.5% versus 35.1%, *P* = 0.02) compared with persons without elevated MH symptoms. The proportion of increased smoking cigarettes was approximately double among persons with elevated depression symptoms (59.4% versus 33.3%, *P* = 0.01), elevated PTS symptoms (65.4% versus 33.3%, *P* = 0.01), and “any elevated MH symptom” (60.5% versus 30.9%, *P* < 0.01). There were no statistically significant differences found between reduced exercise and any of the MH symptoms. The proportion of any maladaptive health behaviour during this period of the pandemic was greater for persons with elevated MH symptom compared with the persons without elevated MH symptoms for anxiety (46.5% versus 34.8%, *P* = 0.01), depression (48.4% versus 33.8%, *P* < 0.001), PTS (53.1% versus 33.7%, *P* < 0.0001), and “any MH symptom” (47.9% versus 32.7%, *P* < 0.0001).

Since figure 2 summarizes results as being similar across individual MH symptoms, for simplicity, adjusted logistic regression analysis is presented only for “any elevated MH symptoms.” During this period of the pandemic, the odds of “any elevated MH symptoms” were double for persons who increased alcohol use [aOR 2.14, 95%CI (1.50–3.05)], four times greater for males who increased marijuana use [aOR 4.18, 95%CI (1.18–14.74)], almost four times greater for females who increased smoking cigarettes [aOR 3

.68 95% CI (1.15–11.86)], and nearly double for any maladaptive health behaviour [aOR 1.93 95% CI (1.44–2.60)] compared with the persons without “any elevated MH symptoms” (Table 2).

**Table 2.** Adjusted logistic regression analysis, for “any elevated mental health symptom” (anxiety, depression, or PTS) predicted by maladaptive health behaviours, stratified by gender.

Maladaptive health behaviours during COVID-19	*N*	Any elevated mental health symptoms (anxiety, depression, or PTS)				
		Overall	*N*	Males	*N*	Females
Increased alcohol use	183	**2.14 (1.50–3.05)**	63	**1.99 (1.09–3.63)**§	120	**2.12 (1.36–3.32)**
Increased marijuana use	53	**2.31 (1.16–4.64)**	19	**4.18 (1.18–14.74)**§	34	1.44 (0.57–3.60)§
Increased CBD use	36	1.82 (0.60–5.48)	15	6.22 (0.68–57.21)§	21	1.44 (0.37–5.59)§
Increased cigarette smoking	37	**2.92 (1.15–7.43)**§	7	2.03 (0.26–16.16)§¥	30	**3.68 (1.15–11.86)**§
Reduced exercise	127	1.32 (0.82–2.13)	39	0.84 (0.35–2.04)§	88	1.77 (0.99–3.19)
Any maladaptive behaviour	245	**1.93 (1.44–2.60)**	81	**1.72 (1.04–2.84)**§	164	**2.06 (1.43–2.99)**

Results presented as odds ratio (OR) and 95% confidence intervals (CI).

Models adjusted by: Age ≥ 65 years, gender, disease type (CD as ref. vs. UC, ileoanal pouch), disease duration (0–10 years as ref. vs. 11–30, 30+ years), income change during COVID-19, immune-modifying therapy.

§Models do not include ileoanal pouch due to small sample size.

§¥ Does not include ileoanal pouch or disease duration due to small sample size.

BOLD: Statistically significant at *P* < 0.05.

## Discussion

Worldwide, individuals have been impacted by the COVID-19 pandemic, and this impact may be particularly pronounced for vulnerable persons such as those with IBD.^17^ Our results indicated that the odds of any elevated MH symptoms including anxiety, depression, and/or PTS was double for persons with IBD who experienced any maladaptive health behaviour during the pandemic to date (November 2020). As the distribution of our data was heavily skewed toward women; nearly three-quarters experienced “any elevated MH symptoms,” our findings were presented stratified by gender. Because of the cross-sectional nature of our study, we cannot determine which came first, the maladaptive health behaviour or the MH symptom. However, our findings inform health care providers that when either of increased MH symptoms or maladaptive health behaviours are found in persons with IBD, inquiry into the other should be pursued. Our data reflect a particularly stressful time in the world impacting on all the citizens especially Canadians, and hence, our data might have implications for other jurisdictions where a major environmental stress is occurring.

### Current health behaviours among persons with IBD during the COVID-19 pandemic

Among persons with IBD who reported current alcohol consumption, there were no significant differences between “any elevated MH symptom” and no elevated MH symptoms. The prevalence of current alcohol consumption among persons with IBD (both with/without MH symptoms) was reported as 65% from our study, and is corroborated by a study by Swanson et al.^18^ The proportion of current marijuana/CBD users and cigarette smokers were greater among the elevated MH group, comparable to other studies.^19,20^ We found the proportion of persons with IBD who regularly exercised since COVID-19 was 20% lower among persons with “any elevated MH symptoms” compared with persons without “any elevated MH symptoms.” Lower levels of physical activity among persons with IBD might be due to disease barriers during the non-COVID periods (GI symptoms, bathroom access, symptom exacerbation). Though there were no significant differences found among those who reported exercising less during the pandemic compared with the pre-pandemic times and MH, our survey question regarding “regularly exercising since COVID-19” provides a grey area. Participants may have responded based on how much they exercised during the pandemic. Hence, the lower prevalence of physical activity found among those with IBD and MH symptoms may in part be related to social isolation in light of physical distancing mandates.

### Increase in health behaviours (maladaptive) among persons with IBD during the COVID-19 pandemic

We defined increase in any of alcohol, marijuana, CBD, cigarette smoking, and reduced exercise during the first wave up until November 2020 of the COVID-19 pandemic as “negative” or maladaptive health behaviours.

#### Alcohol

Persons with IBD who increased alcohol consumption during the COVID-19 pandemic had double the odds of “any elevated MH symptom.” Alcohol consumption was the only health behaviour whose prevalence was not different between MH groups but was reported as increased during the pandemic. This is concerning because of the evidence supporting worsening of gastrointestinal symptoms following alcohol consumption in persons with IBD.^18^ These findings have yet to be replicated, yet the evident impact from the pandemic on social, economic, and health implications for global population resulting in a dramatic rise in the consumption of alcohol play a suggestive role in the current findings. Among respondents who reported that they increased their alcohol, only 44% quantified by how much they increased they increased their alcohol. Hence, our analysis only assesses outcomes among those who increased their alcohol intake versus those who did not. We were not able to quantify changes in outcomes by amounts of increased alcohol intake.

#### Marijuana/CBD (Cannabis)

The odds of “any elevated MH symptoms” was significantly greater in males who increased marijuana use during the pandemic and not females. Based on our study, the increased marijuana use during the pandemic was driven by elevated depression and PTS symptoms (fig. 2). There is evidence to support the association between cannabis use and depressive symptoms,^21^ and greater odds of marijuana use found among male patients with IBD^22^ during pre-pandemic times. However, there is a gap in research relating the association between cannabis and MH stratified by gender among persons with IBD. One recent web-based population survey (first wave of the pandemic in Canada) found those with PTSD who increased cannabis use during the pandemic were more prone to undergo worsening of depression symptoms.^23^

Marijuana and CBD have become increasingly popular to patients as a potential way to self-manage their IBD^19^ and could be a contributing factor to the elevated proportions of cannabis use found in this IBD population both with and without MH symptoms. In addition, during this time period of the COVID-19 pandemic, research on barriers to health care services for those suffering with chronic diseases^24,25^ suggest that people with IBD may have relied more heavily on these “alternative treatments” when there were challenges in accessing more traditional care for their health condition(s).

## Cigarette smoking

Increased cigarette smoking during the pandemic in females and not males with IBD was associated with an increased odds of clinically significant MH symptoms. A study assessing the impact of the COVID-19 pandemic on smoking habits in persons with IBD during the same time period found; frequency of and preoccupation with anxiety was associated with increased smoking during the COVID-19 pandemic.^26^ That more of our non-respondents were known to be smokers based on Registry data we may have underestimated the prevalence of smoking during the pandemic, and hence not captured the full impact of the pandemic on increased smoking.

Worldwide people have greatly been affected by the COVID-19 pandemic, and psychosocial impacts have been reported as acute panic, anxiety, obsessive behaviours, depression, and PTSD in the long run. Though this is not generalized among persons with IBD, it’s implied by the results of this study that the COVID-19 pandemic increased the likelihood of MH symptoms, and the odds were potentially heightened by an increase in maladaptive health behaviours. However, we cannot discern what came first, and hence, MH symptoms may have preceded maladaptive health behaviours. It is important for health care providers to recognize this association. Promotion of adaptive health behaviours such as physical activity among this population is essential, particularly during the higher stress times such as the pandemic. Health care providers should be proactive in inquiring about MH and finding appropriate referrals. Specific strategies on how to provide education to patients around the relationship between maladaptive health behaviours and MH during high-stress times should be considered.

Our study has several limitations. The participants were skewed to older females, and hence, comparisons across genders were not wholly balanced. Secondly, as a cross-sectional study, we can only report on the association of MH symptoms with maladaptive health behaviours; we cannot discern what came first. Lastly, MH symptoms were defined based on self-report scales for screening MH disorders and maladaptive health behaviours were defined based on a single-response item for each behaviour. Our study has several strengths though. Firstly, our participants were drawn from a population-based registry of persons with IBD. The timing of data collection is also a strength. Survey responses were collected during the second wave of the pandemic, a time where social isolation and economic burden were at their peak. This was a time that the general population and especially persons with a chronic immune disease using immunosuppressive drugs may have felt the most vulnerable.

## Conclusion

Persons with IBD have been greatly impacted by COVID-19. Increases in any of alcohol, marijuana, CBD, cigarette smoking, or reduced exercise doubled the likelihood of clinically significant MH symptoms in persons with IBD. Targeting maladaptive health behaviours and promotion of adaptive health behaviours may be an early intervention strategy to mitigate MH symptomatology. Alternatively, if an increase in MH symptoms predated maladaptive health behaviours then health care providers should be inquiring about MH and providing appropriate referrals.

## Supplementary Material

gwad030_suppl_Supplementary_Table_S1

## Data Availability

The data underlying this article are available in the article and in its online supplementary material.
