# Supplementary material for: The Association Between Increased Maladaptive Health Behaviours and Elevated Mental Health Symptoms Among Persons with IBD During the COVID-19 Pandemic
Source: J Can Assoc Gastroenterol. 2023 Sep 13;6(5):179–85. doi: 10.1093/jcag/gwad030 (PMC10558191; doi:10.1093/jcag/gwad030)
Supplement: gwad030_suppl_Supplementary_Table_S1 [file gwad030_suppl_supplementary_table_s1.docx]

**Supplementary Table S1.** Demographic characteristics for respondents compared to non-respondents.

|  | **Respondents (*n* = 1,363)** | **Non-respondents (*n* = 1,579)** | ***P*-value** |
| --- | --- | --- | --- |
| Mean age, years (SD) | 58.0 (13.9) | 57.9 (14.7) | 0.8141 |
| Male gender *n* (%) | 552 (40.50) | 725 (45.9) | 0.0032 |
| Disease phenotype *n* (%) | — | — | 0.1906 |
| Crohn’s disease | 647 (48.9) | 784 (50.9) | — |
| Ulcerative colitis | 646 (48.8) | 710 (46.1) | — |
| Ileoanal pouch | 30 (2.3) | 47 (3.1) | — |
| Marital status *n* (%) | — | — | <0.0001 |
| Never married | 122 (8.9) | 401 (25.5) | — |
| Married/common-law | 1,074 (79.1) | 1,007 (64.0) | — |
| Widowed/divorced/separated | 162 (11.9) | 165 (10.5) | — |
| Smoking status (%) | — | — | <0.0001 |
| Current smoker | 120 (8.9) | 367 (23.3) | — |
